# Effect of *Glycyrrhiza uralensis* extract, *Lactobacillus acidophilus* and their combined supplementation on production performance, immunity, antioxidation and intestinal health in broilers

**DOI:** 10.3389/fvets.2025.1675593

**Published:** 2025-10-17

**Authors:** JianXiong Lu, ShuaiBing Li, JiaHui Wu, Yan Chen, SuSu Jiang, GuoHua Zhang

**Affiliations:** School of Life Sciences and Engineering, Northwest Minzu University, Lanzhou, China

**Keywords:** *Glycyrrhiza uralensis* extract, *Lactobacillus acidophilus*, production performance, immunity, antioxidation, intestinal health, broiler

## Abstract

This study investigated the effects of *Glycyrrhiza uralensis* extract (GUE), *Lactobacillus acidophilus* (Lac), and their combination on production performance, immune and antioxidant functions, and intestinal health in broilers. A total of 420 one-day-old male *Liangfenghua* broilers were randomly assigned to four groups and fed with basal diet, GUE diet with 0.1% GUE, Lac diet with 4.5 × 10⁷ CFU/kg Lac, or GUE+Lac diet with 0.1% GUE and 4.5 × 10⁷ CFU/kg Lac. The experiment lasted 84 days. The results demonstrated that both GUE and Lac enhanced health by increasing immune organ indices, serum levels of IFN-γ, IL-2, IgA, and IgG, and activities of GSH-Px and SOD (*p* < 0.05). They also improved intestinal morphology and barrier function by increasing villus height and the villus height to crypt depth ratio (VH/CD), upregulating expression of Zonula occludens-1 (*ZO-1*), mucin-2 (*MUC2*), and Occludin (*OCLN*), and reducing expression of *IL-1β*, *TLR4*, and *TNF-α* (*p* < 0.05), which increased nutrient metabolic rates (*p* < 0.05). These changes ultimately resulted in improved production performance, evidenced by increased body weight and decreased abdominal fat rate (*p* < 0.05). Notably, the combined GUE+Lac exhibited positive synergistic effects, leading to enhanced immune function as shown by increased serum levels of IL-2, IFN-γ, IgA, and IgG; improved antioxidant capacity indicated by elevated GSH-Px activity in serum and liver; and enhanced intestinal morphology and barrier function by increased villus height and VH/CD ratio, upregulated expression of *ZO-1*, *MUC2*, and *OCLN*, and reduced expression of *IL-1β* and *TLR4*, *TNF-α* (*p* < 0.05) compared to both GUE and Lac alone. This synergism between GUE and Lac significantly increased body weight and nutrient metabolic rates while decreasing abdominal fat rate in broilers. Altogether, we demonstrated the synergistic enhancement of production performance, immunity, antioxidation and intestinal health in broilers by combining GUE and *Lactobacillus acidophilus*. These findings provide robust scientific support for the future development and application of feed additive.

## 1 Introduction

High human demand for poultry meat has led to intensive production practices. Broilers raised under such a production system are susceptible to external factors including diseases, nutritional, and environmental challenges, which often result in poor health, such as enhanced stress response, and reduced immunity. In recent decades, antibiotic growth promoters have been widely used in the poultry industry to control diseases and enhance growth performance. However, the residues of antibiotics in animal derived foods and the development of drug-resistant bacteria pose health risks to consumers and the poultry industry. Therefore, it is essential to develop feed additives from natural sources such as medicinal plants and probiotics for poultry production that can both increase productive potential and maintain broiler health.

*Glycyrrhiza uralensis* is an herb from the *leguminous* family (1), and its rhizome is primarily used in traditional Chinese medicine (2). *Glycyrrhiza uralensis* extract (GUE) contains over 400 compounds, including triterpenoid saponins (*glycyrrhizic* acid), flavonoid glycosides (liquiritigenin, liquiritin) and polysaccharides. These compounds display diverse pharmacological effects such as anti-inflammatory, antioxidant, antibacterial, and antiviral (3, 4). Studies showed that Glycyrrhizic acid inhibited *Mycoplasma gallisepticum* (MG)-triggered inflammation and apoptosis by suppressing the MAPK pathway in chickens (5). *Glycyrrhiza uralensis* flavonoids reduced colonic myeloperoxidase activity and inflammatory cytokine production in mice with ulcerative colitis, effectively suppressing the inflammatory response (6). *Glycyrrhiza* chalcone A inhibited LPS-induced ROS production in murine monocyte macrophages in a dose-dependent manner (7). *Glycyrrhiza* polysaccharide, as a feed additive, enhanced growth performance and serum immunoglobulin level, increased cecum flora abundance and diversity in broilers (8). Additionally, *Glycyrrhiza* polysaccharide increased intestinal secretory immunoglobulin A (SIgA) level, promoted the secretion of goblet cells, and improved the intestinal barrier function in broilers (9).

Probiotics, a class of microorganisms, promote animal growth and health (10), with *Lactobacillus* being one of the most extensively studied and utilized probiotics in poultry production. *Lactobacillus* used fermentable carbohydrates to produce lactic acid, lowering intestinal pH, inhibiting harmful bacteria growth, improving the structure of intestinal mucosa and absorption of nutrients, which results in an increased growth performance in poultry (11, 12). Research has demonstrated that *Lactobacillus plantarum* enhances the growth performance, serum immunoglobulin content, and cecal flora in broilers (13). *Lactobacillus acidophilus* increased body weight gain and feed conversion ratio in Ross broilers (14). Furthermore, *Lactobacillus* promoted the recovery of intestinal mucosa and preserved the integrity of intestinal epithelial barrier by regulating the expression of tight junction proteins and inflammatory cytokines (15).

GUE, a water-soluble extract of *Glycyrrhiza uralensis* containing various bioactive components, promoted the colonization of beneficial bacteria while inhibiting the growth of harmful bacteria in the gut of broilers through its bioactive substances (16), thereby aiding probiotics like *Lactobacillus* in exerting their effects. Certain plant polysaccharides enhanced the growth of beneficial gut bacteria, while probiotics could also facilitate the absorption and utilization of active plant substances (17). Synbiotic benefits can also be driven by probiotic carbohydrate-metabolizing enzymes, enabling utilization of specific oligosaccharides and enhancing probiotic survival, as shown in epilactose-producing cellobiose 2-epimerase systems (18). Studies have confirmed the effects of *Lactobacillus* on growth performance in fast-growing broilers, as well as the synergistic benefits of synbiotics composed of probiotics and medicinal plants, such as *Astragalus* and their active components (19, 20). Our previous study also demonstrated that GUE effectively mitigated the damage caused by Deoxynivalenol (DON)-Zearalenone (ZEN) contamination (21); both GUE and *Lactobacillus acidophilus* as feed additives enhanced growth performance and improved the balance of intestinal microecology in the cecum, and their combined use showed an even more positive effect in *Liangfenghua* broilers (22), a medium-growing strain popular for its excellent meat quality. This study was conducted to further investigate their effects on carcass trait, meat quality, immune and antioxidant functions, and intestinal health, aiming to understand the mechanisms underlying their promotion of broiler production.

## 2 Materials and methods

The *Glycyrrhiza uralensis* extract (GUE; purity >98%; glycyrrhizin ≥10%; prepared from the root of *Glycyrrhiza uralensis* using water boiling extraction and ethanol precipitation methods; Yalan Pharmaceutical Co, Gansu, China) and the *Lactobacillus acidophilus* (Lac; Zhongxin Bio-Technology Co., Hebei, China; 3 × 10^9^ CFU/g) used in the present study were commercial products. The number of viable *Lactobacillus* cells was quantified using the method described by Zeng et al. (23), and was consistent with product labels.

### 2.1 Ethics statement

All procedures involving in animals were performed following the Regulations for the Administration of Affairs Concerning Experimental Animals (Ministry of Science and Technology, China, 2004) and were approved and supervised by the Northwest Minzu University Animal Care and Use Committee (Permit No. xbmu-sm-20230513).

### 2.2 Experimental design, diets and management

A total of 420 healthy one-day-old male *Liangfenghua* broilers were randomly divided into 4 groups, each with 7 replicates of 15 chickens, with no significant difference in initial body weight (40.26 ± 0.83) across groups. The broilers were allocated to the following dietary treatments: a basal diet (CON group), basal diet supplemented with 0.1% GUE (GUE group) (22, 24) basal diet supplemented with Lac at 4.5 × 10⁷ CFU/kg (Lac group), and basal diet supplemented with 0.1% GUE and 4.5 × 10^7^ CFU/kg Lac (GUE+Lac group). The basal diet was formulated according to the broiler Feeding Standard in China (NY/T 33-2004) and was detailed in Table 1.

**Table 1:** Composition and nutrient contents of the basal diets (on air-dry basis).

Items	Content	
	Starter period (day1–28)	Grower-finisher period (day 29–84)
Ingredients (%)		
Corn	55.00	57.48
Soybean oil	2.90	4.20
Soybean meal	29.00	24.00
Cottonseed meal	1.50	1.70
Rapeseed meal	2.18	2.80
Corn gluten meal	6.90	6.60
CaHPO_4_	1.80	2.50
NaCl	0.30	0.30
*L*-Lysis·HCL	0.15	0.11
*DL*-Met		0.08
Cys	0.07	0.03
Premix^a^	0.20	0.20
Total	100.00	100.00
Nutrient levels (%)^b^		
ME, MJ/kg	12.49	12.86
CP	21.46	19.91
Ca	0.90	0.86
Total P	0.68	0.65
Lys	1.15	1.00
Met	0.70	0.40

a The premix provided the following per kg of diets: 1–28 days of age, VA 12000 IU, VD_3_ 3,500 IU, VE 60 IU, VK_3_ 4 mg, VB, 2.5 mg, VB_1_ 10 mg, VB_6_ mg, VB_12_ 8 μg, D-pantothenic acid 40 mg, nicotinic acid 75 mg, folic acid 10 mg, biotin 0.8 mg, choline 700 mg, Zn 90 mg, Fe 110 mg, Cu 20 mg, Mn 100 mg, I 0.5 mg, Se 0.3 mg. 29 to 84 days of age, VA 10000 IU, VD_3_ 3,000 IU, VE 50 IU, VK_3_ 3.5 mg, VB_1_ 2 mg, VB_2_ 10 mg, VB_6_ 5 mg, VB_12_ 6 μg, D-pantothenic acid 20 mg, nicotinic acid 60 mg, folic acid 8 mg, biotin 0.6 mg, choline 600 mg, Zn 80 mg, Fe 100 mg, Cu 15 mg, Mn 80 mg, I 0.5 mg, Se 0.3 mg.

b ME was calculated value, and the other nutrient levels were all measured values.

The trial was conducted at Gansu Agricultural Vocational Farm Co., Gansu, China. The chickens were housed in 3-layer ladder-type cages from 1 to 28 days old, and the nursery was preheated before the birds were introduced. The temperature inside the nursery was maintained at 34 °C with a relative humidity (RH) of 50% during the first week, gradually decreasing by 2 °C each week until the coop temperature reached 26 °C with RH at 45%. From 29 to 84 days old, the broilers were raised on the ground with the coop temperature at 26 °C and RH at 45%, ensuring the coop remained dry, hygienic, and well-ventilated. All the broilers were kept in a single room comprising four floor pens, each measuring 400 × 500 cm. Each pen had solid white plastic walls and was divided by wire mesh into 7 compartments. These compartments were equipped with a round feeder pan (diameter = 50 cm) and one nipple drinker. Cork shavings were used as bedding and were replaced every 3 days. The experiment lasted 84 days. During the test period, chickens had ad libitum access to feed and water. They were vaccinated with the Newcastle disease vaccine and the infectious bursal disease polyvalent vaccine at 7 and 14 days of age, respectively (21).

### 2.3 Sample collection and immune organ indices

On the last day of the starter stage (days 1–28), the grower stage (days 29–56), and the finisher stage (days 57–84), that was on day 28, 56 and 84 of the experiment, after a 12-h fasted feeding, 2 broilers were randomly selected from each replicate, with a total of 14 broilers per group for sampling. 5 mL of blood samples were collected from the wing vein of each individual, and serum was obtained by centrifuging at 3,000 rpm for 10 min. After that, the broilers were euthanized by severing the jugular vein and dissected immediately, and the thymus, bursa of Fabricius, and spleen were collected and weighed. The ratio of organ weight to body weight was used to determine relative organ weights (g/kg); approximately 5 g samples of liver, and breast muscle were collected; approximately 5 cm of intestinal segments from the middle of the jejunum (from the most distal insertion point of the duodenal mesentery to the junction with Meckel’s diverticulum) were excised. The jejunum samples were gently rinsed with ice-cold phosphate-buffered saline (PBS; pH 7.4) and split into two segments. One segment was immediately fixed in 10% paraformaldehyde solution for paraffin section, and the other was longitudinally cut and the mucosa gently scraped into a sterile tube using a sterilized glass slide for RT-PCR.

### 2.4 Apparent metabolic rate of nutrients

At 56 day of age, one broiler was chosen randomly from each replicate and housed in a metabolic cage with a tray for collecting excrement. All excreta were collected at 09:00 and 17:00 daily for three consecutive days. Following the addition of 10% sulfuric acid, the excreta was pooled per broiler and stored in a self-sealing bag at −20 °C until analysis.

The dry matter (DM), crude protein (CP), crude ash (Ash), crude fat (ether extract, EE), and crude fiber (CF) contents in excreta samples and representative dietary samples collected were determined using the standard procedures of the Association of Official Analytical Chemists (AOAC; 2007), after which their apparent metabolic rates were calculated.

### 2.5 Carcass traits

On day 84, two broilers with a body weight close to the average in each replicate were selected and killed after fasting for 12 h. Following bleeding and plucking, the carcass was individually weighed. Subsequently, the birds were eviscerated, and eviscerated carcass weights were measured. The half-eviscerated carcass weight was calculated by excluding the trachea, esophagus, intestines, spleen, pancreas, gallbladder, reproductive organs, and gizzard contents and corneum from the carcass. The eviscerated weight was calculated by removing the heart, liver, proventriculus, gizzard, lungs, and abdominal fat from the half-eviscerated carcass. The rates of dressing, half-eviscerated carcass, eviscerated carcass, breast muscle, thigh muscle (thigh and drumstick), and abdominal fat (fat around the abdomen and gizzard) were calculated as relative weight to the live BW, following “Technical specification for performance testing of meat-type chicken” (NY/T828-2004, China). Moreover, meat of the right breast was sampled for meat quality assessment.

### 2.6 Meat quality

The pH at 45 min (pH_45 min_) and 24 h (pH_24 h_) post-mortem were measured at three sites in the breast muscles using a pH meter (HP818M; XIMA Instrument, China). Each sample was analyzed three times at various points, and the average values were used. Meat colors were measured according to the CIE (Commission Internationale de L’Eclairage) system [Hunter-L* (lightness), a* (redness), and b* (yellowness) values] with a colorimeter (CR-18, DOHO Co., Shenzhen, China) after 45-min postmortem. A skinless meat sample with approximately 1 cm thickness and 2.5 cm diameter was cooked to an internal temperature of 70 °C, and shear force was determined using a C-LM3B shear apparatus (Tenovo, Beijing, China). Meat samples with about 1 cm thick and 2.5 cm in diameter were wrapped in a layer of gauze, placed between filter papers, and subjected to a force of 35 kg for 5 min using a determinator (MAEC-18, MINGAO Instrument Co., Nanjing, China). The water loss rate was calculated by comparing the sample weight before and after squeezing, then dividing by the initial sample weight. To determine the cooking rate, meat samples were cut into approximately 3 × 3 cm pieces, steamed in a pot for 30 min, and re-weighed after cooling to room temperature. The cooking rate was calculated by dividing the sample weight after steaming by the initial sample weight.

Approximately 2.0 g meat sample was freeze-dried using a vacuum freeze dryer and then ground to a homogeneous powder. The fat (ether extract, EE) in the sample was extracted using a Soxhlet extractor (SOX406, Hanon Advanced Technology Group Co., Ltd., Shandong, China) (GB 5009.6–2016, China). The protein content was determined by Kjeldahl method using Kd780 Kjeldahl Nitrogen Analyzer (Peiou Analytical Instrument Co., Ltd., Shanghai, China). The contents of crude fat and crude protein are expressed as percentages in the fresh samples, respectively.

### 2.7 Biochemical and immune indices in serum

The activities of alanine aminotransferase (ALT), aspartate aminotransferase (AST) and alkaline phosphatase (ALP), and the contents of total cholesterol (TC), triglyceride (TG), high-density lipoprotein cholesterol (HDL-C), low-density lipoprotein cholesterol (LDL-C), very low density lipoprotein cholesterol (VLDL-C) and urea nitrogen (UN) in serum were determined using an automatic biochemical analyzer (URIT-8021, Guilin Ulit Medical Electronic Co, Guilin, China).

The levels of interleukin-2 (IL-2), interferon-γ (IFN-γ), and immunoglobulin A and G (IgA and IgG) in serum were measured using commercial ELISA kits (Solarbio Science & Technology Co., Beijing, China) according to the manufacturer’s instructions.

### 2.8 Antioxidant indices in serum and liver

Nine volumes of cold saline was added to 0.5 g of liver sample, which were ground in a glass homogenizer to prepare a tissue homogenate. The homogenate was centrifuged at 2000 rpm for 15 min, and the supernatant was stored at −20 °C. The activities of glutathione peroxidase (GSH-Px) and superoxide dismutase (SOD), and malondialdehyde (MDA) content in the supernatant and serum were measured using commercial assay kits (Servare Bioetechnology Co., Wuhan, China) according to the manufacturer’s instructions.

### 2.9 Jejunum morphological analysis

Jejunum samples fixed in paraformaldehyde were embedded in paraffin, sliced, dehydrated, and stained with hematoxylin and eosin. The histological changes in the jejunum were observed using a CX22 light microscope (OLMPUS, Tokyo, Japan) (24). Three fields were selected for each section, which contained 8 to 10 intact villi in each view. Images were captured using an Olympus microsystem (Tokyo, Japan), and the determination of villi height and crypt depth was performed using Image-Pro Plus image analysis software. Subsequently, the ratio of villus height to crypt depth (VH/CD) was calculated.

### 2.10 Real-time PCR analysis

Total RNA was extracted from the jejunum mucosa using RNAiso Plus Kit (Accurate Biology, Changsha, China). The purity and concentration of RNA were determined using Nano drop One (Thermo, America). When the A260/A280 ratio of RNA falled within the range of 1.8–2.1, it was used to synthesize cDNA. The cDNA was synthesized with a reverse transcription kit (TaKaRa Biotechnology, Beijing, China) following the manufacturer’s instruction. The primer sequences used in Real-time fluorescence quantitative PCR were listed in Table 2. Amplification was performed in a total volume of 10 μL containing 5 μL of SYBR Green PCR Master Mix (TaKaRa Biotechnology, Beijing, China), 0.4 μL of each primer, 1 μL of cDNA, and 3.2 μL of ddH_2_O. The reaction conditions were as follows: denaturation at 95 °C for 30 s; followed by 40 cycles of 95 °C for 5 s, 60 °C for 30 s, and 72 °C for 30 s. The normalization stability of *β-actin* gene expression was tested, confirming it as an endogenous gene suitable for normalizing the target gene data. The relative fold changes in the expression of target genes were calculated by the 2^−ΔΔCt^ method, with *β-actin* serving as the internal control gene to normalizing target gene expression.

**Table 2:** Primer sequences for real-time quantitative PCR.

Gene	Accession number	Primer sequences (5′ to 3′)	Product size (bp)
Interleukin-1β (*IL-1β*)	XM-015297469.3	F: ACTGGGCATCAAGGGCTA	131
		R: GGTAGAAGATGAAGCGGGTC	
Tumor necrosis factor-α (*TNF-α*)	XM-015294124.4	F: GAGCGTTGACTTGGCTGTC	64
		R: AAGCAACAACCAGCTATGCAC	
Toll-like receptor 4 (*TLR4*)	NM-001030693.2	F: ATGCCCAGCAGAGCGGCTCCCACC	178
		R: CGATTCTCACTCAAATCTACAACCT	
Zonula occluden-1 (*ZO-1*)	XM-015278980.4	F: CTTCAGGTGTTTCTCTTCCTCCTC	131
		R: CTGTGGTTTCATGGCTGGATC	
Occludin (*OCLN*)	NM-205128.1	F: ACGGCAGCACCTACCTCAA	123
		R: GGGCGAAGAAGCAGATGAG	
Mucin-2 (*MUC2*)	XM-040673077.2	F: TCCTACAAAAGCACCTAGCACA	102
		R: ACAACTTCACGGCCACTTCTCA	
*β-actin*	NM-205518.2	F: TCCACCGCAAATGCTTCTAA	104
		R: AAGCCATGCCAATCTCGTGT	

### 2.11 Statistical analysis

The data were analyzed using SPSS software version 25.0 (IBM Corp., NY, USA). A two-way ANOVA (using the General Linear Model, GLM) was conducted to analyze the main effects of the GUE and Lac factors and their interaction. One-way analysis of variance (ANOVA) and post-hoc comparisons (Duncan’s test) were performed to assess differences among the GUE, Lac, and GUE+Lac groups. GraphPad Prism 9.0 (GraphPad Software Inc., CA, USA) was utilized for data visualization. Data variation was expressed as standard error of the mean (SEM), and the significance level was set at *p* < 0.05.

## 3 Results

### 3.1 Carcass characteristics and meat quality

The effects of the supplements on carcass traits and meat quality on d 84 were presented in Table 3. Broilers fed diets with GUE, Lac, and GUE+Lac showed a significantly higher live body weight (BW) and evisceration rate (*p* < 0.05), and a lower abdominal fat rate (*p* < 0.05) compared to the CON group. However, no significant differences (*p* > 0.05) were observed in the other traits tested. Furthermore, the combined GUE+Lac group showed significantly higher BW and lower abdominal fat rate compared to both the GUE and Lac groups, exhibiting a significant interaction effect on these measures (*p* < 0.05).

**Table 3:** Effects of GUE, Lac, and their combined supplementation on carcass characteristics and meat quality of broilers at 84 days of age (%).

Items	Group				SEM	Main effect				*p*-value		
						GUE		Lac				
	CON	GUE	Lac	GUE+Lac		−	+	−	+	GUE	Lac	GUE ×Lac
BW	3398^c^	3554^b^	3539^b^	3781^a^	27.21	3,469	3,668	3,476	3,660	0.029	0.035	0.003
DR	85.32	85.67	86.35	85.16	1.23	85.84	85.42	85.50	85.76	0.875	0.922	0.733
HER	75.21	77.98	79.69	78.40	0.97	77.45	78.19	76.60	79.05	0.101	0.330	0.511
ER	63.61^b^	66.49^a^	67.37^a^	66.97^a^	0.92	65.49	66.73	65.05	67.17	0.043	0.021	0.046
BMR	11.65	13.12	12.93	12.03	0.64	12.29	12.58	12.39	12.48	0.334	0.862	0.745
LMR	13.91	14.22	13.85	12.36	0.51	13.88	13.29	14.07	13.11	0.088	0.096	0.101
AFR	2.19^a^	1.88^b^	1.94^b^	1.53^c^	0.36	2.07	1.71	2.04	1.74	0.026	0.045	0.001
pH_45 min_	6.23	6.06	6.28	6.10	0.08	6.26	6.08	6.15	6.19	0.087	0.623	0.678
pH_24 h_	5.52	5.50	5.51	5.58	0.05	5.52	5.54	5.51	5.55	0.913	0.812	0.096
L^*^	45.68	47.01	45.30	45.72	0.96	45.49	46.37	46.35	45.51	0.086	0.789	0.159
a^*^	2.51^b^	3.22^a^	2.97^a^	2.98^a^	0.39	2.74	3.10	2.87	2.98	0.030	0.047	0.091
b^*^	11.90	11.18	11.39	11.07	0.72	11.65	11.13	11.54	11.23	0.631	0.689	0.761
Shear force, N	30.80	30.75	30.76	30.54	1.61	30.78	30.65	30.78	30.65	0.156	0.356	0.624
Cooking rate	67.50	69.02	68.40	69.97	1.63	67.95	69.50	68.26	69.19	0.096	0.103	0.215
Water loss rate	33.75	32.79	29.91	32.55	1.89	31.83	32.67	33.27	31.23	0.363	0.101	0.133
Crude fat	4.07	3.89	3.95	3.93	0.51	4.01	3.91	3.98	3.94	0.614	0.421	0.223
Crude protein	22.59	23.04	22.76	23.13	1.36	22.68	23.09	22.82	22.95	0.543	0.441	0.356

Data represent the mean value (*n* = 14) for each treatment group. CON, control; GUE, *Glycyrrhiza uralensis* extract; Lac, *Lactobacillus acidophilus*; SEM, Standard error of the mean. BW, Live body weight; DR, dressing rate; HER, half-eviscerated rate; ER, eviscerated rate; BMR, breast muscle rate, LMR, Leg muscle rate; AFR, abdominal fat rate; −, not add; +, add. ^a,b,c^ Values with the same or no letter superscripts in the same row mean no significant difference (*p* > 0.05), while with different letter superscripts mean significant difference (*p* < 0.05).

There were no significant differences (*p* > 0.05) in pH_45 min_, pH_24 h_, lightness (L*) value, yellowness (b*) value, shear force, cooking rate, water loss rate, crude fat and crude protein contents of breast meat in broilers among the groups. Likewise, the GUE+Lac treatment did not yield reciprocal effects (*p* > 0.05). However, the redness (a*) values of breast meat was significantly higher (*p* < 0.05) in the GUE, Lac and GUE+Lac groups compared to CON group, although no interaction effect in the GUE+Lac group was observed (*p* > 0.05). These results suggested that both GUE and Lac supplementation enhanced growth and improved carcass quality, and their combined application had a synergistic effect, while having a limited effect on meat quality in broilers.

### 3.2 Apparent metabolic rate of nutrients

Compared to the CON group, the apparent metabolic rates of CP, EE, and CF in broilers significantly increased (*p* < 0.05) by 9.56, 3.18, and 6.45% with GUE; 7.31, 6.34, and 10.32% with Lac; and 13.53, 8.47, and 20.97% with GUE+Lac, respectively (Table 4). Notably, the combined GUE+Lac group exhibited the highest apparent metabolic rates of CP, EE, and CF, with a positive interactive effect (*p* < 0.05).

**Table 4:** Effects of GUE, Lac, and their combination on apparent metabolic rates of nutrients in broilers (%).

Items	Group				SEM	Main effect				*p*-value		
						GUE		Lac				
	CON	GUE	Lac	GUE+Lac		−	+	−	+	GUE	Lac	GUE ×Lac
DM	70.83	69.59	72.31	71.46	1.04	71.57	70.53	70.21	71.89	0.551	0.246	0.416
CP	52.42^c^	57.43^b^	56.25^b^	59.51^a^	0.75	54.34	58.47	54.93	57.88	0.032	0.040	0.011
EE	72.88^c^	75.20^b^	77.50^c^	79.05^a^	0.45	75.19	77.13	74.04	78.28	0.056	0.043	0.002
CF	26.36^c^	28.06^b^	29.08^b^	31.89^a^	0.33	27.72	29.98	27.21	30.49	0.061	0.023	0.001
Ash	30.06	29.37	30.40	31.19	0.36	30.23	30.28	29.72	30.80	0.891	0.765	0.089

Data represent the mean value (*n* = 7) for each treatment group. CON, control; GUE, *Glycyrrhiza uralensis* extract; Lac, *Lactobacillus acidophilus*; SEM, Standard error of the mean; DM, dry matter; CP, crude protein; EE, crude fat; CF, crude fiber; Ash, crude ash; −, not add; +, add. ^a,b,c^ Values with the same or no letter superscripts in the same row mean no significant difference (*p* > 0.05), while with different letter superscripts mean significant difference (*p* < 0.05).

### 3.3 Immune organ indices

Lac supplementation increased‌ (*p* < 0.05) ‌the bursa index‌ at 28 days and spleen index‌ at 56 days. ‌Supplementary GUE and GUE+Lac ‌significantly increased‌ ‌the indices of thymus and bursa of Fabricius‌ at 28 days and ‌that of spleen‌ at 56 days, ‌and the combined application GUE+Lac exhibited a positive interactive effect‌ (*p* < 0.05). No significant differences‌ (*p* > 0.05) ‌were observed‌ in these indices at 84 days among groups (Table 5).

**Table 5:** Effect of GUE, Lac and their combined supplementation on immune organ indices of broilers (g/kg).

Items	Group				SEM	Main effect				*p*-value		
						GUE		Lac				
	CON	GUE	Lac	GUE+Lac		−	+	−	+	GUE	Lac	GUE ×Lac
Day 28												
Thymus	4.92^b^	5.26^a^	5.08^b^	5.34^a^	0.12	5.00	5.30	5.09	5.21	0.044	0.111	0.033
Spleen	1.15	1.20	1.19	1.29	0.08	1.17	1.25	1.18	1.24	0.101	0.556	0.126
Bursa	2.09^b^	2.34^a^	2.38^a^	2.56^a^	0.10	2.24	2.45	2.22	2.47	0.043	0.049	0.041
Day 56												
Thymus	5.51	5.54	5.58	5.67	0.11	5.55	5.61	5.53	5.63	0.788	0.626	0.239
Spleen	1.27^b^	1.40^a^	1.47^a^	1.49^a^	0.10	1.37	1.45	1.34	1.48	0.026	0.042	0.044
Bursa	1.30	1.38	1.31	1.39	0.09	1.31	1.39	1.34	1.35	0.880	0.965	0.155
Day 84												
Thymus	3.33	3.44	3.39	3.54	0.09	3.36	3.49	3.39	3.47	0.411	0.551	0.175
Spleen	1.12	1.11	1.14	1.22	0.07	1.13	1.17	1.12	1.18	0.961	0.789	0.841
Bursa	0.80	0.90	0.91	0.94	0.10	0.86	0.92	0.85	0.93	0.231	0.452	0.110

Data represent the mean value (*n* = 14) for each treatment group. CON, control; GUE, *Glycyrrhiza uralensis* extract; Lac, *Lactobacillus acidophilus*; SEM, Standard error of the mean; −, not add; +, add. ^a,b,c^ Values with the same or no letter superscripts in the same row mean no significant difference (*p* > 0.05), while with different letter superscripts mean significant difference (*p* < 0.05).

### 3.4 Serum biochemical parameters

The activity of ALT, AST, and ALP, as well as the content of HDL-C and UN in the serum of 28- and 84-day-old broilers, were not affected (*p* > 0.05) by any of the supplements. However, TC, TG and VLDL-C content in 28- and 84-day-old broilers and LDL-C content in 84-day-old broilers were significantly reduced (*p* < 0.05) by the supplements (Table 6). The GUE+Lac group showed a interactive effect (*p* < 0.05), leading to a significant elevation in serum LDL-C and TC levels compared to the other groups.

**Table 6:** Effects of GUE, Lac, and their combined supplementation on serum biochemical parameters of broilers.

Items	Group				SEM	Main effect				*p*-value		
						GUE		Lac				
	CON	GUE	Lac	GUE+Lac		−	+	−	+	GUE	Lac	GUE ×Lac
Day 28												
ALT, U/L	3.67	4.33	4.00	3.67	0.29	3.84	4.00	4.00	3.84	0.904	0.899	0.363
AST, U/L	165	161	163	162	11.33	164	162	163	163	0.101	0.922	0.863
ALP, U/L	539	531	521	522	16.66	530	526	535	521	0.343	0.453	0.746
UN, mmol/L	0.78	0.89	0.81	0.70	0.05	0.80	0.80	0.84	0.76	0.334	0.521	0.200
TG, mmol/L	1.53^a^	1.35^b^	1.40^b^	1.32^b^	0.06	1.47	1.34	1.44	1.36	0.038	0.560	0.068
TC, mmol/L	4.71^a^	4.45^b^	4.27^b^	3.93^c^	0.29	4.49	4.19	4.58	4.10	0.026	0.041	0.076
VLDL-C, mmol/L	0.82^a^	0.69^b^	0.71^b^	0.65^b^	0.02	0.77	0.67	0.76	0.68	0.041	0.049	0.141
HDL-C, mmol/L	1.76	1.70	1.82	1.78	0.08	1.79	1.74	1.73	1.80	0.524	0.860	0.148
LDL-C, mmol/L	3.19^a^	2.97^a^	2.87^a^	2.49^b^	0.29	3.03	2.73	3.08	2.68	0.065	0.043	0.045
Day 84												
ALT, U/L	4.33	4.00	4.33	4.17	0.41	4.33	4.09	4.17	4.25	0.228	0.167	0.154
AST, U/L	221	216	233	230	8.72	227	223	219	231	0.507	0.528	0.262
ALP, U/L	453	447	427	441	16.53	440	444	450	434	0.801	0.727	0.459
UN, mmol/L	0.86	0.70	0.87	1.00	0.08	0.87	0.85	0.78	0.94	0.730	0.127	0.077
TG, mmol/L	1.70^a^	1.51^b^	1.53^b^	1.48^b^	0.05	1.62	1.50	1.61	1.51	0.023	0.033	0.107
TC, mmol/L	4.57^a^	4.03^b^	3.91^b^	3.64^c^	0.17	4.24	3.84	4.30	3.78	0.016	0.031	0.001
VLDL-C, mmol/L	0.96^a^	0.73^b^	0.81^b^	0.71^b^	0.03	0.89	0.72	0.85	0.76	0.045	0.031	0.191
HDL-C, mmol/L	1.79	1.80	1.84	1.74	0.11	1.82	1.77	1.80	1.79	0.887	0.938	0.564
LDL-C, mmol/L	2.85^a^	2.58^b^	2.44^b^	2.33^c^	0.17	2.65	2.46	2.72	2.39	0.045	0.033	0.011

Data represent the mean value (*n* = 14) for each treatment group. CON, control; GUE, *Glycyrrhiza uralensis* extract; Lac, *Lactobacillus acidophilus*; SEM, Standard error of the mean; −, not add; +, add; ALT, activities of alanine aminotransferase; AST, aspartate aminotransferase; ALP, alkaline phosphatase; UN, urea nitrogen; TG, triglyceride; TC, total cholesterol; VLDL-C, very low density lipoprotein cholesterol; HDL-C, high-density lipoprotein cholesterol; LDL-C, low-density lipoprotein cholesterol. ^a,b,c^ Values with the same or no letter superscripts in the same row mean no significant difference (*p* > 0.05), while with different letter superscripts mean significant difference (*p* < 0.05).

### 3.5 Immune and inflammatory factors in serum

**Figure 1:**
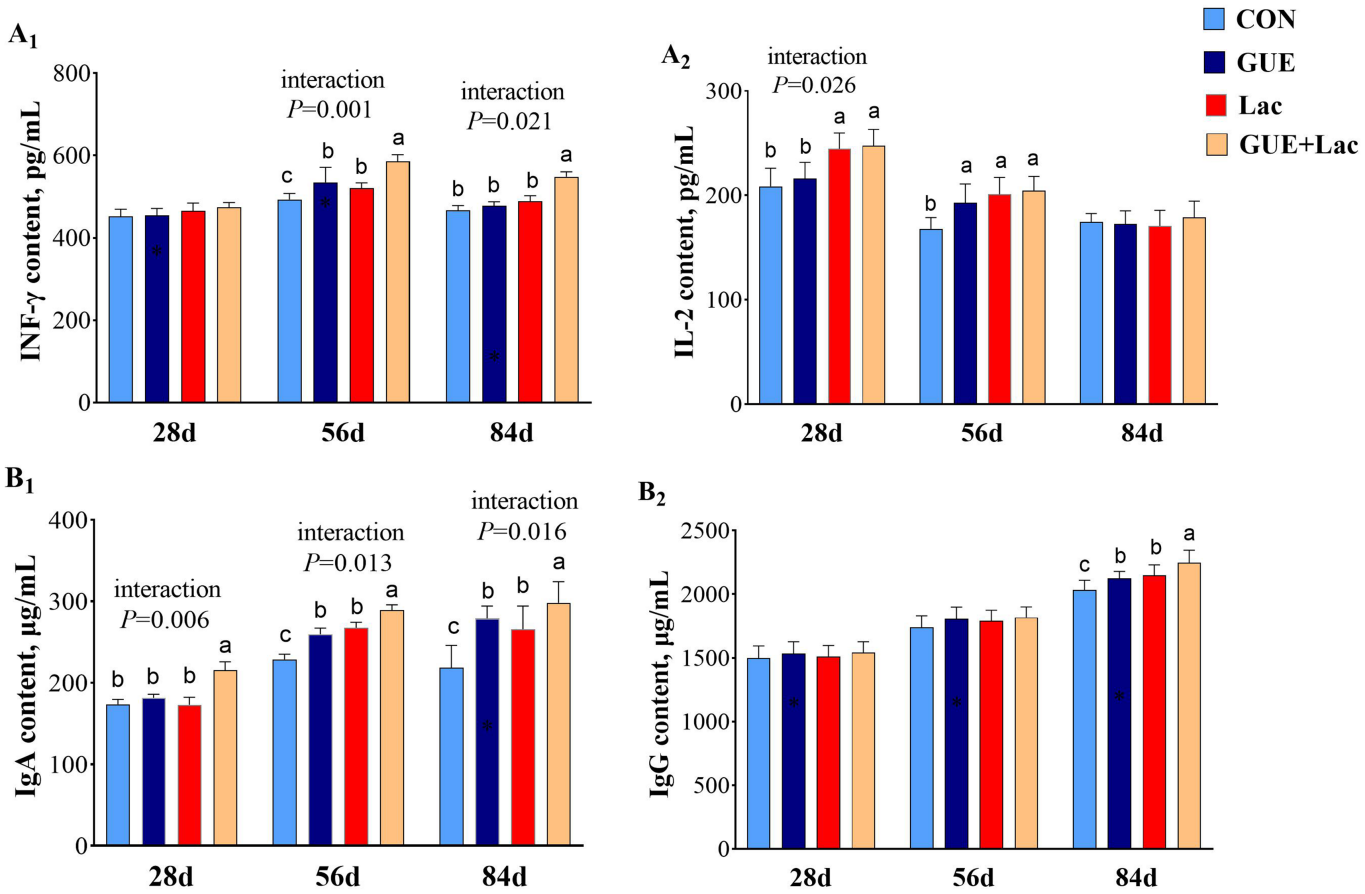
Effects of GUE, Lac, and their combined supplementation on serum immune parameters. CON: control; GUE, *Glycyrrhiza uralensis* extract; Lac, *Lactobacillus acidophilus*; IFN-γ, interferon-γ; IL-2, interleukin-2; IgA, immunoglobulin A; IgG, immunoglobulin G. Data are presented as mean ± SD (*n* = 14). ^a,b,c^Means denoted by different superscripts are significantly different (*p* < 0.05).

The levels of INF-γ and IgG in the serum of 28-day-old broilers were not affected (*p* > 0.05) by the supplements (Figure 1). However, Lac significantly increased IL-2 content, and the combined application of GUE+Lac exhibited a positive interactive effect, leading to increased IL-2 and IgA content compared to the CON group (*p* < 0.05). At 56 days of age, the serum content of INF-γ, IL-2, and IgA in broilers was significantly increased (*p* < 0.05) by the supplements. Additionally, the GUE+Lac treatment demonstrated a significant interaction effect, resulting in higher INF-γ and IgA levels compared to the other groups (*p* < 0.01). By 84 days of age, both GUE and Lac significantly increased (*p* < 0.05) the levels of IgA and IgG, and the GUE+Lac group showed a positive interactive effect, with the highest INF-γ, IgA and IgG levels among all groups (*p* < 0.05).

### 3.6 Serum and liver antioxidant parameters

**Figure 2:**
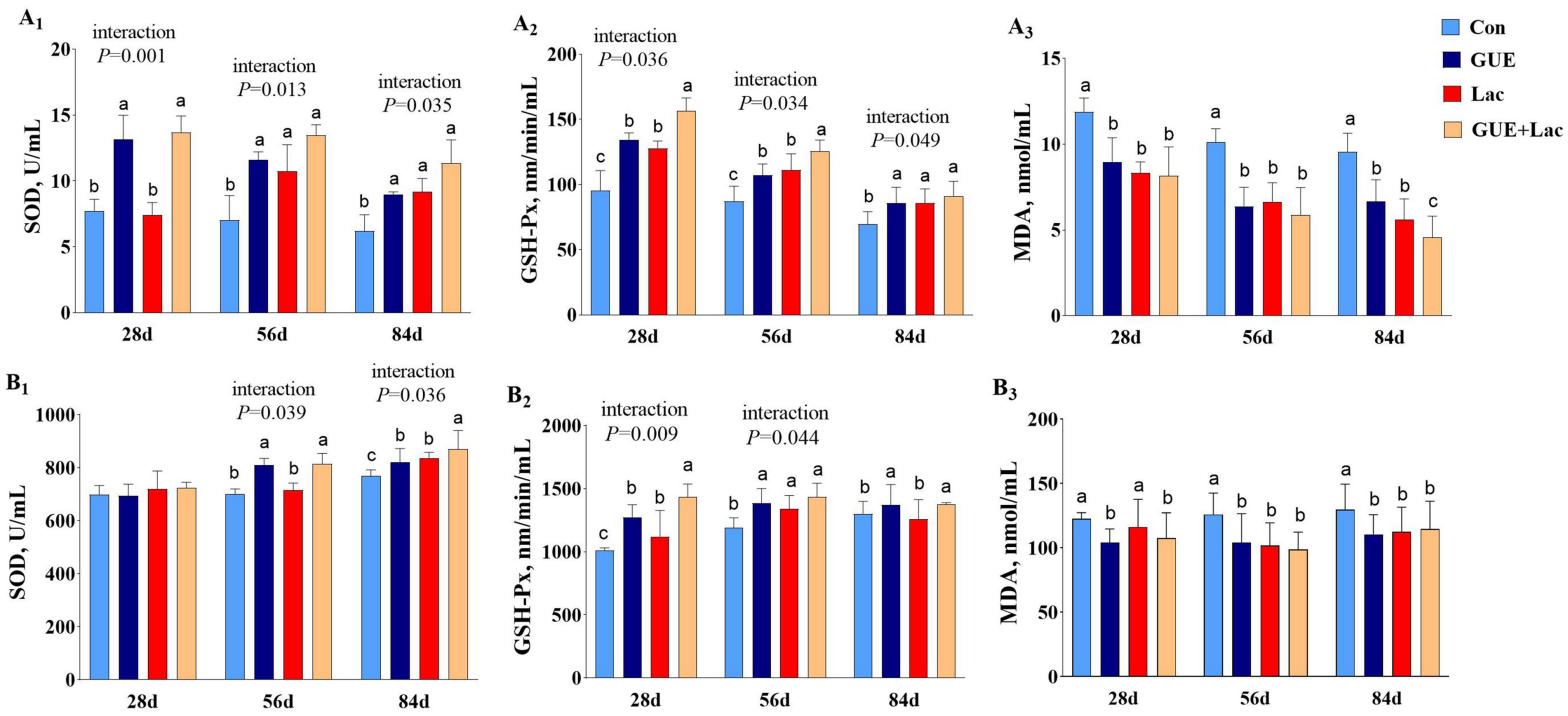
Effects of GUE, Lac, and their combined supplementation on serum (A_1_–A_3_) and live (B_1_–B_3_) antioxidant parameters. CON, control; GUE, *Glycyrrhiza uralensis* extract; Lac, *Lactobacillus acidophilus*; SOD, superoxide dismutase; GSH-Px, glutathione peroxidase; MDA, malondialdehyde. Data are presented as mean ± SD (*n* = 14). ^a,b,c^Means denoted by different superscripts are significantly different (*p* < 0.05).

Compared to the CON group, both the GUE and GUE+Lac groups significantly increased (*p* < 0.05) SOD and GSH-Px activities and reduced MDA content in the serum across all three age groups. Lac significantly increased (*p* < 0.05) the SOD and GSH-Px activities and reduced serum MDA content, but had no significant effect on SOD at day 28 (*p* > 0.05). Additionally, the GUE+Lac group showed an interactive effect on SOD and GSH-Px activities, resulting in significantly elevated GSH-Px activities at day 28 and 56 (*p* < 0.05) (Figure 2A_1_–A_3_). The effects of supplements on liver antioxidant indices in broilers were presented in Figure 2B_1_–B_3_. Both GUE and GUE+Lac supplementation significantly increased (*p* < 0.05) the activities of SOD and GSH-Px, while decreasing MDA content in the liver across all three age groups, but had no significant effect on SOD activity at day 28 (*p* > 0.05). Lac supplementation increased (*p* < 0.05) GSH-Px activity at day 28 and 56, SOD activity at day 84, and decreased (*p* < 0.05) MDA content at day 56 and 84. Additionally, the GUE+Lac group showed an interactive effect on GSH-Px at day 28, SOD and GSH-Px at day 28, and SOD at day 84, leading to significantly elevated activities of GSH-Px at day 28 and SOD at day 84 compared to the GUE and Lac groups (*p* < 0.05).

### 3.7 Intestinal morphology in the jejunum

**Figure 3:**
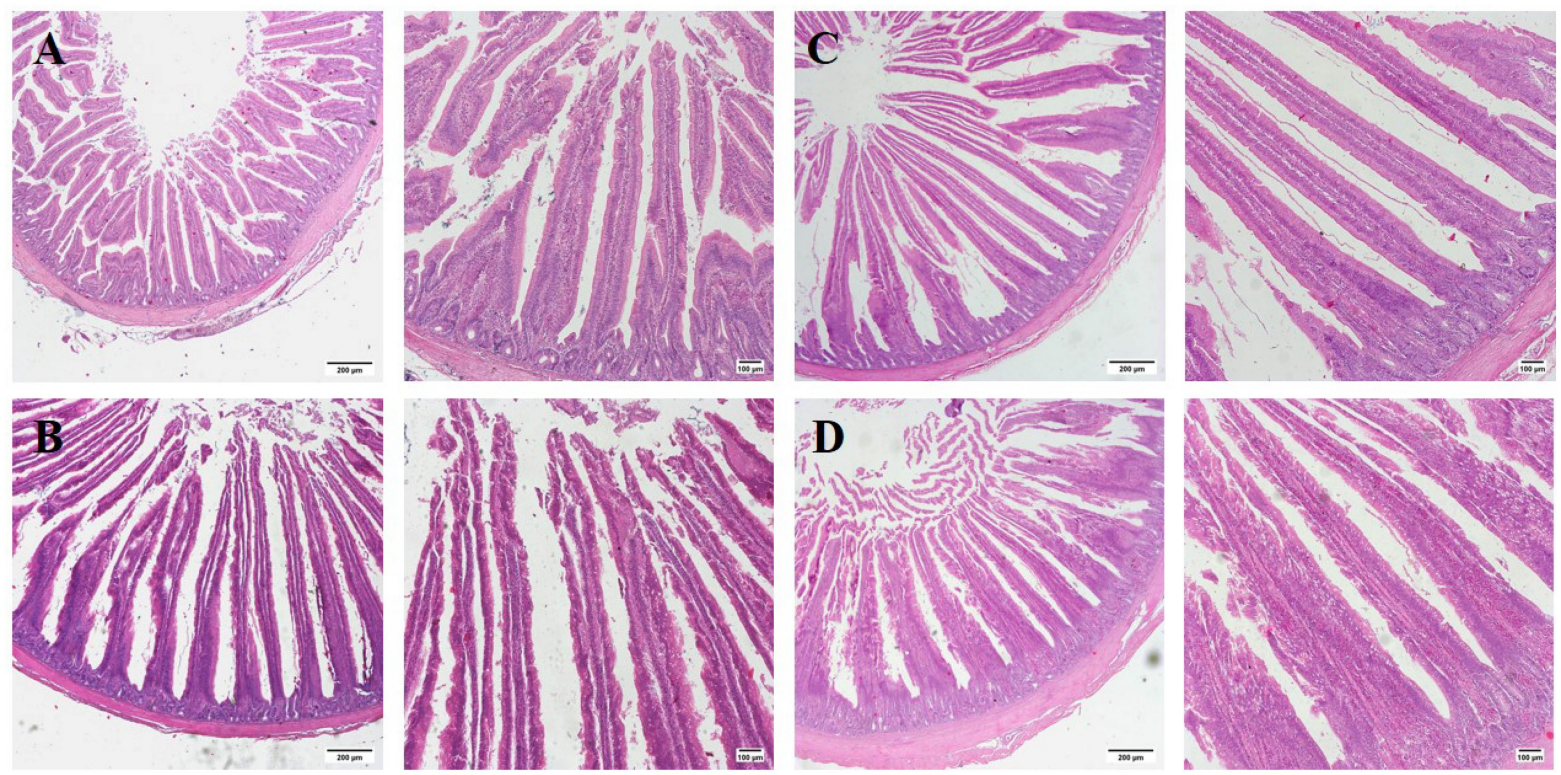
Histological representations of the H and E-stained jejunal sections of broilers at 28 days of age. **(A)** CON group; **(B)** GUE group; **(C)** Lac group; **(D)** GUE+Lac group. Scale bars represent 200 μm and 100 μm, respectively.

The supplements significantly increased (*p* < 0.01) the villus height and the ratio of villous height to crypt depth (VH/CD) in the jejunum of broilers at day 28, 56, and 84, with no significant effect (*p* > 0.05) on the crypt depth (Figure 3; Table 7). Furthermore, the GUE+Lac group significantly increased (*p* < 0.01) the villus height and VH/CD at day 28 and 56 compared to GUE and Lac groups, though no significant interaction effect was observed (*p* > 0.05).

**Table 7:** Effects of GUE, Lac, and their combined supplementation on the jejunal morphology in broilers.

Items	CON	GUE	Lac	GUE+Lac	SEM	*p*-value
Day 28						
Villus height, μm	612.46^c^	663.98^b^	738.66^a^	788.37^a^	7.98	0.013
Crypt depth, μm	116.44	106.59	111.21	103.21	2.63	0.054
VH/CD	5.32^c^	6.44^b^	6.82^b^	7.69^a^	0.16	0.007
Day 56						
Villus height, μm	861.62^c^	985.79^b^	1066.31^b^	1188.09^a^	7.28	0.005
Crypt depth, μm	169.29	162.15	166.53	161.22	2.29	0.110
VH/CD	5.09^c^	6.08^b^	6.40^b^	7.37^a^	0.09	0.011
Day 84						
Villus height, μm	1099.60^b^	1268.00^a^	1222.05^a^	1269.06^a^	9.22	0.060
Crypt depth, μm	201.13	203.94	195.46	191.17	3.14	0.059
VH/CD	5.47^b^	6.22^a^	6.26^a^	6.64^a^	0.10	0.041

Data represent the mean value (*n* = 14) for each treatment group. CON, control; GUE, *Glycyrrhiza uralensis* extract; Lac, *Lactobacillus acidophilus*; SEM, Standard error of the mean; VH/CD, villus height/crypt depth. ^a,b,c^ Values with the same or no letter superscripts in the same row mean no significant difference (*p* > 0.05), while with different letter superscripts mean significant difference (*p* < 0.05).

### 3.8 Expression of inflammatory cytokines and tight junction-related proteins in jejunal mucosa

**Figure 4:**
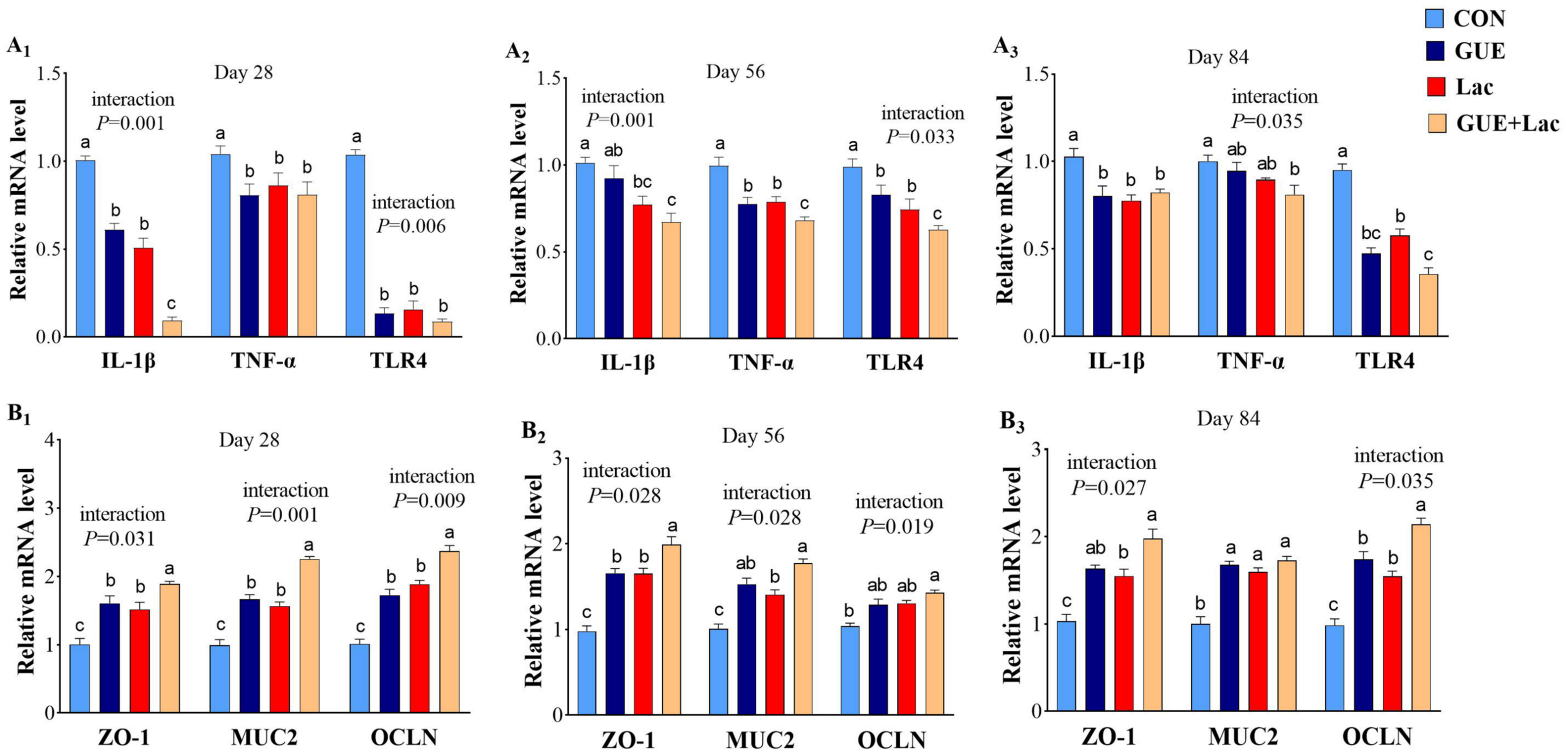
The mRNA expression of inflammatory cytokines (A_1_–A_3_) and tight junction-related proteins (B_1_–B_3_) in the jejunum. CON, control; GUE, *Glycyrrhiza uralensis* extract; Lac, *Lactobacillus acidophilus*; *ZO-1*, zonula occluden-1; *MUC2*, mucin-2; *OCLN*, occludin. Data are presented as mean ± SD (*n* = 14). ^a,b,c^Means denoted by different superscripts are significantly different (*p* < 0.05).

Supplementation of both GUE and Lac reduced the expression of *IL-1β* and *TLR*4 in the jejunal mucosa of broilers at 28, 56 and 84 days, and the *TNF-α* expression at days 28 and 56. GUE+Lac showed an interactive effect, leading to further decreased the expression of *IL-1β* at day 28 and *TNF-α* and *TLR4* at day 56 compared to the GUE and Lac groups (*p* < 0.05) (Figure 4A_1_–A_3_). Both GUE and Lac upregulated the expression of *ZO-1* and *MUC2* at days 28, 56 and 84, and the expression of *OCLN* at days 28 and 84 (*p* < 0.05). Additionally, the interaction of GUE+Lac was observed, which increased the expression of *ZO-1* and *OCLN* at days 28 and 84 compared to the GUE and Lac groups (*p* < 0.05) (Figure 4B_1_–B_3_).

## 4 Discussion

Dietary supplementation with GUE increased BWG in broilers reared at high-density in both the grower and total phase (25), as well as FCR of broilers (26). *L. acidophilus* boosted feed efficiency and immune function in broilers by regulating nutrient metabolism and inflammation responses. Our previous study showed that supplementation with both GUE and *L. acidophilus* increased the ADG in grower-finisher phase, with their combined use yielding a more favorable effect in *Liangfenghua* broilers (22). Building on these findings, we conducted further investigations into the effects of GUE and *Lactobacillus acidophilus*, particularly, their combined supplementation on carcass performance, immunity, antioxidant capacity, and intestinal health in broilers.

### 4.1 Carcass characteristics and meat quality

Carcass traits encompass the quality and characteristics of animal carcasses after slaughter, serving as a comprehensive indicator for assessing production performance and economic value. In this study, both GUE and Lac supplementation increased the body weight and eviscerated percentage, and decreased the abdominal fat percentage of broilers. We also observed that GUE had no effects on other meat quality indicators of breast muscle but increased the a* value, indicating a slight improvement in meat color (27). Similarly, dietary GUE reduced abdominal fat rate but had no effect on breast muscle rate in broilers (28, 29). Dietary *L. acidophilus* increased the leg muscle rate and reduced the abdominal fat rate in broilers (30, 31). *Lactobacillus* cultures did not affect the a* and L* values of breast and leg muscle but increased the b* values of leg muscle in broilers (32). These findings suggested that both GUE and *L. acidophilus* supplementation could improve carcass quality to some extent by reducing abdominal fat and increasing total net carcass weight, without negatively affecting meat quality in broilers.

Additionally, both supplementary GUE and *L. acidophilus* increased the apparent metabolic rates of CP, EE, and CF in broilers. These findings indicated that, similar to fast-growing broilers (33), *L. acidophilus* enhanced production performance and feed utilization in medium-growing broilers by improving the digestion, absorption, and utilization of feed nutrients. Previous studies indicated that *Lactobacillus* enhanced the growth performance and the apparent metabolic rate of CP in broilers (20, 34). These effects can be related to the improvement of structure of intestinal flora (34), intestinal morphology, and the abundance of bacteria producing short-chain fatty acids (35). *Glycyrrhiza uralensis* alleviated intestinal inflammation by inhibiting pro-inflammatory factors (e.g., IL-6 and IL-1β) (36), and mitigated ulcerative colitis by repairing mitochondrial damage and increasing SOD, GSH-PX, and IL-10 levels in intestinal epithelial cells (37). These mechanisms collectively maintained the synthesis and activity of digestive enzyme by preventing inflammation and oxidative stress. Accordingly, both GUE and *L. acidophilus* supplementation could improve the production performance of broilers by promoting the digestion and absorption of nutrients (38).

While previous studies have explored the combined use of probiotics and medicinal plant extracts, the effects of their feeding effects appeared to be influenced by various factors, including bacterial strains, bioactive substances in medicinal plants, and the species and growth stages of animals. For example, *Astragalus* and *L. plantarum* together enhanced the growth performance of broilers (19), whereas the combination of complex probiotics with *Astragalus* polysaccharide did not show a similar effect in growing-finishing pigs (39). Our study revealed that the combination of GUE and Lac synergistically promoted the growth and improved the carcass traits by enhancing nutrient digestion and absorption and decreasing abdominal fat rate in broilers. The integration of plant extracts with probiotics enhanced the medicinal effects of plant extracts by leveraging the catabolic properties of probiotics (40). GUE increased the cecal abundance of *Lactobacillus gallinarum* and *Lactobacillus reuteri* in broilers (22). Human intestinal fungus metabolized 18β-glycyrrhetinic acid with low intestinal bioavailability into metabolites with inhibiting the activation of nuclear factor-kappa B (NF-κB) signaling pathway (41). Similarly, probiotic cocktails enhanced the effects of baicalin by accelerating its conversion into highly active compounds in the ileum, thereby increasing the abundance of short-chain fatty acid-producing bacteria in broilers (42).

### 4.2 Blood biochemistry parameters

Serum ALT, AST, and ALP activities reflect liver function, while UN content provides valuable insights into protein metabolism within the body. Our results showed that GUE, Lac and GUE+Lac did not significantly alter ALT, AST and ALP activities, nor UN and HDL-C levels in broiler serum. This indicated that these supplements are safe, do not impair liver function, and have no adverse effects on protein metabolism. In contrast, *L. plantarum* improved liver function by reducing ALT and AST levels in DON-challenged broiler chickens (43), while 18β-glycyrrhetinic acid alleviated DON-induced liver injury by inhibiting nuclear receptor coactivator 4-mediated ferritinophagy and ferroptosis (44).

Serum TG and TC levels serve as important indicators of lipid transport in the body. HDL is involved in cholesterol removal and transport triglycerides back to the liver, LDL is responsible for delivering cholesterol to cells, and VLDL transports triglycerides from the liver to other tissues. These lipoproteins offer an insight into overall lipid metabolism. The *Glycyrrhiza uralensis* supplementation lowered serum TC and TG levels (45), and GUE reduced serum TC and LDL levels in broilers (29). *L. salivarius* decreased the serum TC, LDL-C and TG levels of broilers (46). *Lactobacillus* mixture reduced serum TC and TG levels but did not affect serum HDL-C and LDL-C levels in broiler (34). In this study, both GUE and Lac significantly reduced the serum TC, TG and VLDL-C levels in broilers at days 28 and 84, as well as serum LDL-C level at day 84, while their combination GUE+Lac group showed a interactive effect, leading to a more significant elevation in serum LDL-C and TC levels. This suggested that they promoted cholesterol and lipid metabolism, with their combination proving more effective, which was consistent with the decreased abdominal fat rate in broilers. Although the exact mechanism is not fully understood, previous research suggests that *glycyrrhizic* acid inhibits lipid peroxide formation and facilitates the conversion of cholesterol into bile acids (1), and prevents abnormal lipid metabolism in the liver by modulation of gut microbiota in rats fed a high-fat diet (47). Similarly, plant-derived pectic polysaccharides, such as those from *Rubus chingii* Hu., have been shown to inhibit intestinal lipid absorption *in vivo*, contributing to improved lipid profiles and reduced fat deposition (48). *Lactobacillus* decomposed cholesterol in the digestive tract and lowered serum cholesterol levels (49). It also incorporated cholesterol into bacterial cells and inhibited cholesteryl ester synthesis by reducing the activity of acetyl-CoA carboxylase and 3-hydroxy-3-methylglutaryl-CoA reductase (34).

### 4.3 Immunity

The immune system directly influences animal growth efficiency, metabolic balance, and overall health by defending against pathogens and maintaining physiological homeostasis. The immune organ index reflects the development and functional status of immune organs in animals. Supplementary GUE increased the indices of the thymus and bursa of Fabricius at day 28 and the spleen at day 56, while Lac increased that of bursa of Fabricius at day 28 and the spleen at day 56, suggesting that these supplements could promote the development of immune organs in broiler chickens during early growth. However, both GUE and Lac had no significant effect on the immune organ index at day 84. This suggests that under normal conditions, the immune organs of *Liangfenghua* broilers at day 84 are likely fully developed and no longer influenced by external factors.

Immune factors play a critical role in the regulation of immune responses. Serum levels of IgA and IgG are considered indicators of humoral immunity. Studies showed that GUE enhanced antibody titers against specific and non-specific antigens, thereby improving broilers’ humoral immunity (26). *Glycyrrhiza uralensis* increased serum IgG levels in quail (50) and serum monocyte and granulocyte levels, enhancing innate or specific immune responses in laying hens (51). Some active components in GUE exhibit immune-enhancing and anti-inflammatory effects. *Glycyrrhizic* polysaccharides raised the serum IgA, IgG and IgM levels in broilers (8). *Glycyrrhizic* flavonoids increased the serum IgG content and improved the immune function in piglets (52). Additionally, *L. plantarum* increased serum levels of IgA and IgG in *Daheng* broilers (53) and serum levels of IgA and IgM in Cobb broilers (54). *L. acidophilus* increased the serum IgG level in broilers (33). The probiotic genus *Lactobacillus* exerts positive immunomodulatory effects on multiple immune responses, including cellular immunity, humoral immunity, and intestinal mucosal immunity (55). Our study demonstrates that the inclusion of GUE or Lac in the diet can enhance the broiler’s immunity, evidenced by increased serum levels of IgA at day 56 and IgA and IgG at day 84.

Cytokines have various functions, including regulating immune and participating in inflammatory responses. Serum levels of INF-γ and IL-2 are commonly used as indicators of immune strength (56). INF-γ serves as an immunomodulatory molecule, promoting the maturation of cytotoxic T lymphocytes, B cell proliferation, and antibody production (57). IL-2 enhanced the activities of T cells and stimulated B cells to produce antibodies, serving as a pivotal mediator in immune responses triggered by inflammation (58). Our study observed that GUE increased the serum levels of INF-γ and IL-2 at 56, and Lac increased the serum levels of IL-2 at day 28 and both INF-γ and IL-2 at 56, enhanced humoral immunity and anti-inflammatory capacity in broilers. Likewise, *Glycyrrhizic* polysaccharides raised the increased the immune organ index and serum cytokine IL-2 levels in mice (59). *Glycyrrhizic* flavonoids increased the serum IgG content and improved the immune function in piglets (52). *L. plantarum* increased serum levels of IL-2 and IFN-γ in *Daheng* broilers (53). *L. acidophilus* increased the serum levels of IL-2 and IL-4 in broilers (33).

Furthermore, the combined application GUE+Lac demonstrated a positive interaction effect, resulting in increased serum levels of IL-2 and IgA at day 28, INF-γ and IgA at day 56, and INF-γ, IgA and IgG at 84. These results suggested GUE and *L. acidophilus* could synergistically boost the immunity by stimulating the secretion of immune factors and anti-inflammatory cytokines in broilers. This could be interpreted as *L. acidophilus* accelerating the biotransformation and absorption of the bioactive components in GUE by utilizing the catabolic properties of probiotics, while GUE promotes the proliferation of probiotics, thereby building a mutually reinforcing mechanism. However, further research is needed to fully understand this mechanism.

### 4.4 Antioxidation

The systemic antioxidant capacity is crucial for the health and growth of broilers. An increase in antioxidant capacity is associated with improved immune function and reduced inflammation and oxidative stress. The liver, as a key metabolic organ, is highly susceptible to oxidative stress due to its role in detoxification and biotransformation of xenobiotics that generate reactive oxygen species (ROS). Oxidative stress disrupts redox balance, promoting inflammation and impairing liver function (60). Antioxidant mechanisms included enzymes such as SOD, GSH-Px, and catalase (CAT), along with non-enzymatic molecules like glutathione (GSH), which are essential for neutralizing ROS and maintaining cellular homeostasis (61). Given the persistent challenges posed by oxidative stress, there is an urgent need for exogenous antioxidants that can bolster cellular defenses and support the immune system. GUE, as a natural antioxidant, inhibited mitochondrial lipid peroxidation, scavenged free radicals, and enhanced antioxidant enzyme activity through its active ingredients (62). GUE supplementation enhanced growth performance and increased the activities of SOD, CAT (63) and GSH-Px, while reducing MDA content in serum of broilers (64). *Glycyrrhiza* polysaccharide raised the serum levels of GSH and SOD in broilers, while simultaneously reducing the MDA level (65). Dietary GUE has also demonstrated benefits in enhancing liver antioxidant capacity of broilers. GUE reduced hepatic triglycerides and oxidative stress markers in broilers, as shown by decreased plasma cholesterol and improved lipid profiles (64). In this study, GUE significantly increased the activities of SOD and GSH-Px while decreasing MDA content in the serum and liver of broilers, indicating that GUE enhanced the systemic and hepatic antioxidant capacity by activating the antioxidant enzyme system, thereby alleviating oxidative stress and its harmful effects on health.

We also observed that Lac increased GSH-Px and SOD activities while reducing MDA content in the serum of broilers. It also enhanced hepatic GSH-Px activity at day 28 and 56, and SOD activity at day 84, while decreasing MDA content. These results indicated the beneficial effects of *L. acidophilus* supplementation on systemic and hepatic antioxidant capacity in broilers. Similarly, studies found that *Lactobacillus casei* effectively increased the levels of T-AOC and SOD while reducing the MDA level in broilers (66). *Lactobacillus reuteri* mitigated diquat-induced hepatic oxidative stress and inflammation in hens (67). *Lactobacillus plantarum* upregulated the expression of the nuclear factor erythroid 2-related factor 2 (Nrf2) pathway in the liver, a key regulator of antioxidant responses (68). *Lactobacillus* decreased hepatic MDA level and increased total antioxidant capacity (TAC) by upregulating SOD and GSH-Px in broilers (69). However, Yu et al. (54) reported that dietary *Bacillus coagulans*, but not *Lactobacillus plantarum*, increased activities of GSH-Px and SOD, and decreased MDA level in the serum of broilers challenged by lipopolysaccharide. This indicates that *Lactobacillus*, as a probiotic, has the potential to act as a natural antioxidant in broilers; however, this effect depends on the strain type.

Additionally, the combination of GUE and Lac demonstrated significant interaction effects on SOD and GSH-Px, elevating serum GSH-Px activity at days 28 and 56, as well as hepatic GSH-Px activity at day 28 and SOD activity at day 84. GUE contains various bioactive components that can exert antioxidant and anti-inflammatory effects through multiple shared pathways. *Glycyrrhetinic* acid suppressed the expression of inflammatory factors by blocking NF-κB signaling pathway (70). *Glycyrrhiza* polysaccharides promoted the maturation and cytokine IL-12 secretion of dendritic cells (DCs) through toll-like receptor 4 (TLR4) and down-stream p38 and NF-κB signaling pathways (71). *Glycyrrhizin* maintained intracellular redox balance and eliminated mitochondrial ROS by enhancing the activities of SOD, CAT, and GSH-Px through downregulating high-mobility group box-1 protein (HMGB1)/TLR5 signaling (72). It also reduced inflammatory mediators such as NF-κB p65, IL-1β, and IL-18 by suppressing the HMGB1/TLR4 pathway (73). *Glycyrrhiza* flavonoids affected the levels of SOD, CAT, MDA, and inflammatory factors TNF-α, IL-6, and IL-1β by activating the PI3K-Akt signaling pathway (74). Furthermore, the bioactive components of *Glycyrrhiza uralensis* can undergo biotransformation by microorganisms to produce derivatives with enhanced pharmacological activity (41, 75). 18 β-*glycyrrhetinic* acid, a metabolite of *glycyrrhetinic* acid, exhibited pronounced inhibitory activity on the production of intracellular nitric oxide (76). Therefore, it can be inferred that supplementary Lac and intestinal probiotics, such as *Lactobacillus* augmented by GUE (22), enhanced the biotransformation and utilization of bioactive components of GUE (41), which synergistically improved antioxidant and anti-inflammatory capacity in broilers.

### 4.5 Intestinal health

Our previous study found that both GUE and *L. acidophilus* supplementation enhanced gut antioxidant capacity, promoted sIgA secretion, and modulated the cecal microbial community composition (22). Additionally, GUE effectively mitigated the damage to growth performance and intestinal health caused by deoxynivalenol (DON) and zearalenone (ZEN) contamination in broilers (21). In this study, we observed that supplementation with GUE and Lac significantly increased villus height, the VH/CD ratio in the jejunal mucosa of broilers. The intestinal villi serve as the primary site for nutrient absorption, and the structural integrity of the intestinal morphology significantly influences animal health. Increased villus height provide a larger the absorptive area, promoting nutrient uptake and optimizing production performance in broilers (77).

The intestinal barrier permits the uptake of essential nutrients and immune sensing while restricting pathogenic molecules and bacteria. Both structural and molecular components of the intestinal tract work together to perform this complex yet essential function. The integrity of the intestinal epithelium acts as a physical barrier against enteric pathogen invasion and ensures optimal nutrient absorption (78). Tight junctions (TJs) are the core structures that maintain intestinal barrier function, primarily composed of key proteins such as OCLN and ZO-1. MUC2 is the main component of the intestinal mucus layer, serving as the first physical and chemical barrier of the intestinal defense system. Upregulated TJ proteins improved barrier function and permeability, thereby reducing inflammation and oxidative stress (79). Our result indicated both GUE and Lac upregulated the mRNA expression of of *ZO-1* and *MUC2* in the jejunal mucosa at days 28, 56 and 84, as well as the expression of *OCLN* at days 28 and 84. Thus, the intestinal barrier function was enhanced. Several studies have documented the effects of *Glycyrrhiza uralensis* and its active components on the intestinal health of animals. Ibrahim et al. (16) found that GUE promoted the expression of junctional adhesion molecule 2 (*JAM-2*) and *MUC-2* in the jejunal mucosa of broilers. *Glycyrrhiza* polysaccharide increased the villus height, the VH/CD ratio, and the expression of *Ocludin*, *Claudin-1*, and *MUC2* in the mucosa of jejunum, ileum, and duodenum of broilers (8). *Glycyrrhiza* flavonoids improved the morphological structure of the intestine by increasing villus height and VH/CD in duodenum in piglets (80). Additionally, *L.acidophilus* has been shown to enhance intestinal health and protect against enteric pathogen invasion by increasing the VH/CD ratio and the expression of intestinal barrier function proteins in broilers (65, 81). *L. plantarum* enhanced the digestive enzyme secretion and increased nutrient digestibility by the increasing VH/CD ratio (82), and improved intestinal barrier function and permeability by upregulating the expression of *MUC2*, *Occludin*, and sIgA in the jejunum of broilers (83). These results suggest a promoting effect of GUE and Lac on intestinal development and functional improvement under normal conditions, consistent with the increased nutrient metabolism rate.

Cytokines are critical in regulating intestinal immune function and maintaining immune balance. GUE reduced the expression of *TLR4* and *IL-1β* in the jejunal mucosa of broilers (16), and improved the microstructure of colonic mucosa and reduced the expression of *TNF-α* and *IL-6* in rats (84). *Glycyrrhiza* chalcone inhibited *TNF-α*, *IL-1β*, and *IL-6* expressions in the colon tissue of mice with colitis (85). Furthermore, *L. acidophilus* alleviated inflammatory responses by downregulating the expression of inflammatory cytokine *IL-1β* and *IL-8* in the jejunum of broilers infected with *Clostridium perfringens* (81). In acute colitis mice, *L. acidophilus* reduced intestinal inflammation by suppressing the production of pro-inflammatory cytokine IL-6, TNF-α and IL-1β in colonic tissue (86). In this study, both GUE and Lac reduced the expression of *IL-1β* and *TLR*4 in the jejunal mucosa of broilers at 28, 56 and 84 days, and the *TNF-α* expression at days 28 and 56. This suggested that under normal conditions, GUE and Lac can improve the intestinal immune function in broilers.

The combined supplementation with GUE and Lac increased the villus height and VH/CD at days 28 and 56 compared to GUE and Lac groups. Furthermore, the interaction between GUE and Lac further upregulated expression of *ZO-1* and *OCLN* at days 28 and 84, and decreased the expression of *IL-1β* at day 28 and *TNF-α* and *TLR4* at day 56. This finding suggests that the selected combination of GUE and *L. acidophilus* more effectively enhances the intestinal morphology and promotes intestinal health in broilers. As mentioned previously, the combination could establish a mutually reinforcing mechanism: the active components in GUE promote the growth of probiotics, while the probiotics, in turn, enhance the host’s absorption and utilization of these active substances by improving intestinal barrier function and permeability. This aligns with evidence that microbial metabolites, such as butyric acid, regulate intestinal barrier integrity by modulating post-translational modifications of epithelial proteinsepithelial protein, including GAPDH lactylation and butyrylation, thereby influencing tight junction stability (87).

## 5 Conclusion

In conclusion, dietary supplementation with both GUE and *Lactobacillus acidophilus* enhanced systemic and hepatic antioxidant capacity, immunity, and intestinal health, while improving production performance by increasing digestion and absorption of feed nutrients and eviscerated carcass weight and reducing the abdominal fat rate in broilers. The combined use of GUE and *Lactobacillus acidophilus* showed significant interaction effects, further enhancing the production and health in broilers by building a mutually reinforcing mechanism. These findings demonstrate the promising potential of plant extract-probiotics combinations as feed additives.

## Data Availability

The original contributions presented in the study are included in the article/supplementary material, further inquiries can be directed to the corresponding author.
